# The Complete Chloroplast Genome of *Euphrasia regelii*, Pseudogenization of *ndh* Genes and the Phylogenetic Relationships Within Orobanchaceae

**DOI:** 10.3389/fgene.2019.00444

**Published:** 2019-05-14

**Authors:** Tao Zhou, Markus Ruhsam, Jian Wang, Honghong Zhu, Wenli Li, Xiao Zhang, Yucan Xu, Fusheng Xu, Xumei Wang

**Affiliations:** ^1^School of Pharmacy, Xi’an Jiaotong University, Xi’an, China; ^2^Royal Botanic Garden Edinburgh, Edinburgh, United Kingdom; ^3^Key Laboratory of Resource Biology and Biotechnology in Western China (Ministry of Education), School of Life Sciences, Northwest University, Xi’an, China

**Keywords:** *Euphrasia regelii*, hemiparasite, chloroplast genome, pseudogenization, phylogenetic analyses

## Abstract

*Euphrasia* (Orobanchaceae) is a genus which is widely distributed in temperate regions of the southern and northern hemisphere. The taxonomy of *Euphrasia* is still controversial due to the similarity of morphological characters and a lack of genomic resources. Here, we present the first complete chloroplast (cp) genome of this taxonomically challenging genus. The cp genome of *Euphrasia regelii* consists of 153,026 bp, including a large single-copy region (83,893 bp), a small single-copy region (15,801 bp) and two inverted repeats (26,666 bp). There are 105 unique genes, including 71 protein-coding genes, 30 tRNA and 4 rRNA genes. Although the structure and gene order is comparable to the one in other angiosperm cp genomes, genes encoding the NAD(P)H dehydrogenase complex are widely pseudogenized due to mutations resulting in frameshifts, and stop codon positions. We detected 36 dispersed repeats, 7 tandem repeats and 65 simple sequence repeat loci in the *E. regelii* plastome. Comparative analyses indicated that the cp genome of *E. regelii* is more conserved compared to other hemiparasitic taxa in the Pedicularideae and Buchnereae. No structural rearrangements or loss of genes were detected. Our analyses suggested that three genes (*clpP*, *ycf2* and *rps14*) were under positive selection and other genes under purifying selection. Phylogenetic analysis of monophyletic Orobanchaceae based on 45 plastomes indicated a close relationship between *E. regelii* and *Neobartsia inaequalis.* In addition, autotrophic lineages occupied the earliest diverging branches in our phylogeny, suggesting that autotrophy is the ancestral trait in this parasitic family.

## Introduction

The chloroplast (cp) is the most important organelle for green plants as it is the place where photosynthesis and carbon fixation occurs. The cp genome is uniparentally inherited and generally has a quadripartite structure consisting of one large single-copy (LSC) region, one small single-copy (SSC) region, and two inverted repeat regions (IRs) of the same length (Bendich, 2004). The cp genome is more conserved than the nuclear and mitochondrial genomes in terms of gene structure and composition (Asaf et al., 2017a). Due to the highly conserved and non-recombinant nature of the cp genome, it has been shown to be a very useful genetic resource for inferring evolutionary relationships at different taxonomic levels (Caron et al., 2000; Cho et al., 2015). Recently, with the advent of next generation sequencing, it has become comparatively easy to sequence the complete cp genome of non-model taxa and infer phylogenetic relationships based on whole plastomes (Ruhsam et al., 2015; Guo et al., 2017; Saarela et al., 2018).

The genus *Euphrasia* (Orobanchaceae) is widely distributed throughout temperate regions of the southern and northern hemispheres, and contains about 458 species and subspecies, most of which occur in the northern hemisphere (Gussarova et al., 2008; Secretariat, 2017; Moura et al., 2018). *Euphrasia* plants are either perennial or annual herbs which mainly parasitise the roots of Gramineae species (Wu et al., 2005; Gussarova et al., 2008). Some species in this genus are used as folk medicine to treat diseases such as blepharitis, conjunctivitis and coughs (Li and Wang, 2003). *Euphrasia* was once included in the tribe Rhinantheae of the Scrophulariaceae but based on molecular data, was moved with all other parasitic plants in this family to Orobanchaceae (Olmstead et al., 2001). Due to frequent autogamy as well as interspecific hybridization and morphological diversity, *Euphrasia* comprises a taxonomically complex group of taxa where species delimitation remains challenging (Vitek, 1998; French et al., 2008; Gussarova et al., 2008).

In China, 11 species of *Euphrasia* are currently recognized which are divided into two sections based on morphological characteristics, namely Sect. *Semicalcaratae* and Sect. *Paradoxae* (Hong et al., 1998). The annual herb *Euphrasia regelii* Wettst., belongs to Sect. *Semicalcaratae*, and is used for the treatment of hyperglycemia, inflammation, hay fever, conjunctivitis, colds, influenza and coughs (Shuya et al., 2004). Due to the medicinal value of *E. regelii*, research has mainly focused on identifying the effective chemical constituents of this species (Li and Wang, 2003; Shuya et al., 2004). Few studies have been conducted to infer the phylogenetic position of *E. regelii* or its genetic diversity due to a lack of informative genetic markers. Additionally, research has been hampered because *E. regelii* is difficult to distinguish from other *Euphrasia* species due to morphological similarities. Therefore, more discriminating genetic markers are needed to infer the phylogenetic relationship of *E. regelii* with other *Euphrasia* taxa and to facilitate reliable genetic authentication of this important medicinal herb. Although the cp genome of some Orobanchaceae species has been sequenced and utilized in phylogenetic studies (Wicke et al., 2013; Samigullin et al., 2016; Zeng et al., 2017), no cp genome which could have been used for the development of new and variable markers has been published for the genus *Euphrasia* until now.

In this study, we characterize the complete cp genome of *E. regelii* and compare it with the available cp genomes of Orobanchaceae taxa. Our results will be useful for marker development, species discrimination, and the inference of phylogenetic relationships in the genus *Euphrasia.*

## Materials and Methods

### Plant Material and DNA Extraction

*Euphrasia regelii* was collected from Taibai mountain (107°16′47.172″ E, 33°59′27.1068″ N) in the Chinese province of Shaanxi. Young leaves were put into silica gel for DNA extraction and a voucher specimen was deposited at the herbarium of Xi’an Jiaotong University (XJTU) (Xi’an, China). Total genomic DNA was extracted using a modified CTAB protocol (Doyle, 1987), and the quantity and quality of the extracted DNA was determined by gel electrophoresis and a NanoDrop 2000 Spectrophotometer.

### Chloroplast Genome Sequencing and Assembly

The DNA Library with an insert size of 270 bp was constructed using TruSeq DNA sample preparation kits and sequenced on an Illumina HiSeq X Ten platform with an average paired end read length of 150 bp. The raw reads were filtered to obtain high-quality reads by removing adapters, low-quality sequences such as reads with unknown bases (“N”), and reads with more than 50% low-quality bases (quality value ≤ 10) using the NGS QC Toolkit v2.3.3 (Patel and Jain, 2012). To filter reads from the chloroplast genome, paired-end high quality reads were mapped to the previously published cp genomes (NC_034308, NC_027838, NC_022859, KF922718, NC_022859; Supplementary Table S1) in the Orobanchaceae using Bowtie v2.2.6 with default parameter (Langmead and Salzberg, 2012). Matched paired-end reads were *de novo* assembled using SPAdes v3.6.0 (Bankevich et al., 2012), and the longest contig was selected as Seed sequence for further assembly using NOVOPlasty v2.6.2 (Dierckxsens et al., 2017). Finally, all the clean reads were mapped to the unannotated cp genome using Geneious v10.1 with bowtie 2 algorithm (Biomatters, Ltd., Auckland, New Zealand) in order to avoid assembly errors. Seven regions with low coverage were Sanger sequenced (Supplementary Table S2). The cp genome was aligned to its reverse complement to determine inverted repeat regions. The boundaries of the inverted repeats and single copy regions were also verified by Sanger sequencing (Supplementary Table S2).

### Genome Annotation, Codon Usage, and Repeat Structure

The complete cp genome was annotated using the automatic annotator DOGMA (Wyman et al., 2004) with manual verification via BLAST searches against the cp genomes of other Orobanchaceae species. During the annotation process, open reading frames (ORFs) that can be matched with known cp genes were annotated, and the remaining ORFs lacking protein evidence were disregarded. Genes that contained one or more frameshift mutations or premature stop codons were considered potential pseudogenes. The circular annotated plastid genome map was drawn using the online program OrganellarGenome DRAW (Lohse et al., 2013) and deposited in GenBank (MK070895). The codon usage frequency was calculated based on protein-coding genes using MEGA v6 (Tamura et al., 2013). Tandem repeat sequences were searched for using the Tandem Repeats Finder program (Benson, 1999) with the following parameters: 2 for the alignment parameter match and 7 for mismatch and indels. Dispersed and palindromic repeats were identified using REPuter with a minimum repeat size of 30 bp and sequence identity of no less than 90% (hamming distance equal to 3) (Kurtz et al., 2001). Simple sequence repeats (SSRs) were identified using the software MISA (Thiel et al., 2003) with the following minimum number of repeats: 10 for mono, 5 for di-, 4 for tri-, and 3 for tetra-, penta, and hexa-nucleotide SSRs.

### Genome Comparison and Sequence Divergence

Eleven plastome sequences, including two from non-parasitic taxa (*Rehmannia glutinosa*, NC_034308; *Lindenbergia philippensis*, NC_022859), three from facultative hemiparasites (*Triphysaria versicolor*, KU212369 *Aureolaria virginica*, MF780870; *Buchnera americana*, MF780871), four from obligate hemiparasites (*Neobartsia inaequalis*, KF922718; *Schwalbea americana* NC_023115; *Castilleja paramensis*, NC_031805; *Pedicularis cheilanthifolia*, NC_036010; *Striga aspera*, MF780872) and one from a holoparasite (*Lathraea squamaria*, NC_027838), were retrieved from GenBank and used in the subsequent analyses. Comparative Genomics of 12 Orobanchaceae plastomes was performed and visualized using the mVISTA software (Frazer et al., 2004) with the annotation of *R. glutinosa* as a reference. Any large structural changes such as gene order rearrangements were recorded using Mauve v1.1.1 with default settings (Darling et al., 2004). IR expansion/contraction of these plastomes were also analyzed. The nucleotide diversity (*Pi*) and sequence polymorphism of Rhinantheae species were analyzed using DNAsp v6.0 (Rozas et al., 2017). In order to detect whether plastid genes were under selection, the non-synonymous (dN), synonymous (dS), and dN/dS values of 64 protein coding gene from Rhinantheae species were calculated using the PAML package v 4.0 with YN algorithm (Yang, 2007). Nucleotide substitution rates were not calculated for pseudogenes due to the existence of premature stop codons.

### Phylogenetic Analysis

To infer phylogenetic relationships within Orobanchaceae a total of 42 cp genomes were used with *Salvia miltiorrhiza* (Lamiaceae), *Tectona grandis* (Lamiaceae), and *Solanum lycopersicum* (Solanaceae) as outgroup (Supplementary Table S1). All cp genome sequences were aligned using MAFFT v7.402 (Katoh and Standley, 2013) and the most variable positions were excluded from the alignment using Gblocks v0.91b (Talavera and Castresana, 2007). A maximum likelihood (ML) and a Bayesian inference (BI) approach were used to infer phylogenetic relationships. The Maximum likelihood analyses were conducted using IQ-TREE v1.6.1 (Nguyen et al., 2015) with the best best-fit model selected by ModelFinder and 1,000 bootstrap replicates. Bayesian inference was conducted using MrBayes v3.2.6 (Ronquist et al., 2012) with a nucleotide substitution model inferred by Modeltest v3.7 (Posada and Crandall, 1998) (Supplementary Table S3). The Markov chain Monte Carlo (MCMC) algorithm was run for 1 million generations and sampled every 100 generations. The first 25% of resultant trees were discarded and the remaining trees were used to build a majority-rule consensus tree with posterior probability (PP) values for each node. As gene loss from the cp genome is a common phenomenon in the parasitic family of Orobanchaceae, the most conserved regions (TMCRs) of the cp genomes were retrieved using HomBlocks (Bi et al., 2018). TMCRs were then used to construct the phylogenetic trees using the two methods specified above. Additionally, the phylogeny of the genus *Euphrasia* was inferred using the following chloroplast regions: *trn*L, *trn*L-*trn*F, and *atp*B-*rbc*L. The sequences of 39 *Euphrasia* species were downloaded from TreeBase with the Accession No. 22492^1^.

## Results

### The Chloroplast Genome of *Euphrasia regelii*

**FIGURE 1:**
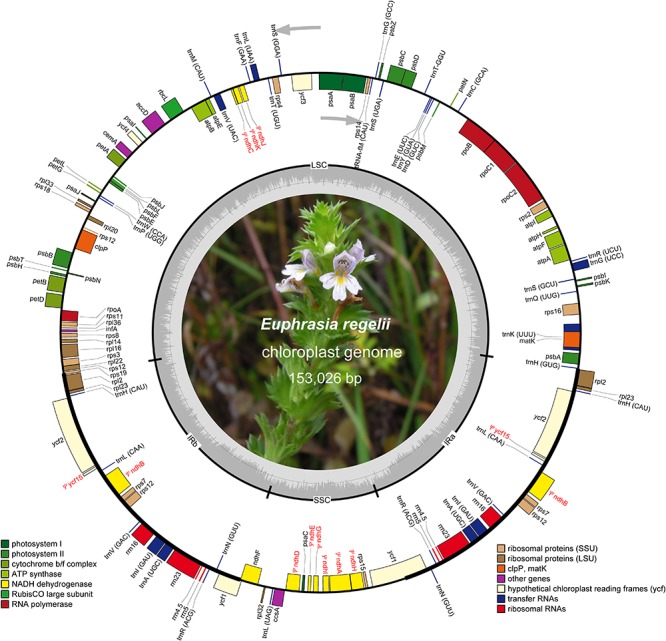
Map of the chloroplast genome of *Euphrasia regelii.* Genes belonging to different functional groups are highlighted in different colors. Gray arrows indicate the direction of gene transcription. Small single copy (SSC), large single copy (LSC), and inverted repeats (IRa, IRb) are indicated. Pseudogenes are marked by Ψ.

A total of 7,867,077 paired-end reads were retrieved with a sequence length of 150 bp. A total of 7,861,321 of high quality reads were used for the cp genome assembly. The raw reads were deposited in NCBI SRA database under the Accession No. SRR8237421. Based on a combination of *de novo* and reference guided assemblies, the cp genome of *E. regelii* was obtained with the average coverage of 956×. The complete cp genome of *E. regelii* is 153,026 bp in length and possesses the typical quadripartite structure including a LSC region of 83,893 bp separated from the 15,801 bp long SSC region by two inverted repeats (IRs), each 26,666 bp (Figure 1 and Table 1).

**TABLE 1:** Statistics of the chloroplast genomes of *Euphrasia regelii* and seven other Orobanchaceae species.

	*E. regelii*	*L. squamaria*	*N. inaequalis*	*S. americana*	*T. versicolor*	*L. philippensis*	*R. glutinosa*	*A. fasciculatum*
Genome length (bp)	153,026	150,504	151,349	160,910	152,448	155,103	153,622	106,796
LSC length (bp)	83,893	81,981	83,806	84,756	83,650	85,606	84,605	43970
SSC length (bp)	15,801	16,061	16,327	6,517	17,520	17,885	17,579	530
IR length (bp)	26,666	26,231	25,566	34,818	25,639	25,800	25,719	31148
No. of different genes	107	78	102	108	106	117	121	66
No. of different protein-coding genes	69	46	69	74	73	80	82	28
No. of different tRNA genes (duplicated in IR)	30 (7)	30 (7)	27 (5)	30 (7)	28 (7)	30 (7)	30 (7)	29 (7)
No. of different rRNA genes (duplicated in IR)	4 (4)	4 (4)	4 (4)	4 (4)	4 (4)	4 (4)	4 (4)	4 (4)
No. of genes duplicated in IR	15	14	10	19	15	16	12	17
No. of different genes with introns	16	12	18	17	15	18	18	10
No. of pseudogenes	11	32	0	2	1	4	0	8
GC content (%)	38.4	38.1	37.5	38.1	38.2	37.8	38.0	34.7

The plastome of *E. regelii* was predicted to contain 105 unique genes, including a set of 71 protein-coding genes, 30 tRNA genes and 4 rRNA genes (Table 1 and Supplementary Table S4). Unexpectedly, 10 plastid genes encoding the subunits of the NAD(P)H dehydrogenase complex (*ndh* genes) were pseudogenized, and only the intact ORF of *ndhF* existed. *Ycf15* was also found to be a pseudogene due to an internal stop codon in its ORF frame. Of 105 genes, four protein-coding genes (*rpl2*, *ycf2*, *rpl23*, *rps7*), seven tRNA genes (*trnH-CAU*, *trnL-CAA*, *trnV-GAC*, *trnI-GAU*, *trnA-GAC*, *trnR-ACG*, *trnN-GUU*), and four rRNA genes (*rrn16*, *rrn23*, *rrn4.5*, *rrn5*) were duplicated in the IR regions. Sixteen intron-containing genes were detected in the *E. regelii* cp genome, including seven protein-coding genes and six tRNA genes with one intron, whereas the remaining three protein-coding genes (*clpP*, *rps12*, *ycf3*) had two introns (Table 2). We found that *trnK*-UUU had the largest intron (2,472 bp) and included the gene *matK*. The tRNA gene *trnL-UAA* had the smallest intron (462 bp) (Table 2). The overall GC content of 38.4% of the *E. regeli* cp genome was generally low (LSC, SSC, and IR regions had 36.2, 33.9, and 42.9% GC content, respectively).

**TABLE 2:** Genes with introns in the chloroplast genome of *Euphrasia regeli.*

		Exon I	Intron I	Exon II	Intron II	Exon III
Gene	Location	(bp)	(bp)	(bp)	(bp)	(bp)
*trn*K-UUU	LSC	37	2,472	35		
*rps*16	LSC	40	839	194		
*trn*G*-*UCC	LSC	23	663	48		
*atp*F	LSC	234	687	411		
*rpo*C1	LSC	456	767	1,668		
*ycf*3	LSC	134	698	229	705	153
*trn*L-UAA	LSC	35	462	50		
*trn*V-UAC	LSC	38	582	35		
*clp*P	LSC	71	728	292	627	228
*pet*B	LSC	6	728	642		
*pet*D	LSC	8	765	475		
*rpl*16	LSC	9	865	399		
*rpl*2	IR	394	669	434		
*rps*12*	IR/LSC	114		232	538	26
*trn*I-GAU	IR	37	946	35		
*trn*A*-*UGC	IR	38	812	35		

*rps12 gene is trans-spliced gene with the two duplicated 3′ end exons in IR regions and 5′ end exon in the LSC region.

### Codon Usage Bias of *E. regelii* cp Genome

The frequency of codons in the *E. regelii* cp genome was calculated based on protein-coding genes (Table 3). In total, all genes were encoded by 23,629 codons. We found that leucine was the most frequent amino acid (2,427 codons, 10.27%) and cysteine (265 codons, 1.1%) the least frequent in the cp genome (Table 3). Similar to other angiosperms cp genomes, codon usage in the *E. regelii* plastome was biased toward a high representation of U and A at the third codon position [relative synonymous codon usage values (RSCU) > 1].

**TABLE 3:** Codon–anticodon recognition pattern and codon usage in the *Euphrasia regeli* chloroplast genome.

Codon	Amino acid	Count	RSCU	tRNA	Codon	Amino acid	Count	RSCU	tRNA
UUU	F	882	1.33	*trnF-GAA*	UAU	Y	667	1.62	*trnY-GUA*
UUC	F	441	0.67		UAC	Y	156	0.38	
UUA	L	747	1.85	*trnL-UAA*	UAA	*	42	1.68	
UUG	L	511	1.26	*trnL-CAA*	UAG	*	17	0.68	
CUU	L	510	1.26	*trnL-UAG*	CAU	H	435	1.48	*trnH-GUG*
CUC	L	157	0.39		CAC	H	152	0.52	
CUA	L	330	0.82		CAA	Q	649	1.5	*trnQ-UUG*
CUG	L	172	0.43		CAG	Q	216	0.5	
AUU	I	952	1.48	*trnI-GAU*	AAU	N	927	1.55	*trnN-GUU*
AUC	I	405	0.63		AAC	N	272	0.45	
AUA	I	569	0.89	*trnI-CAU*	AAA	K	1034	1.5	*trnK-UUU*
AUG	M	506	1	*trnM-CAU*	AAG	K	342	0.5	
GUU	V	452	1.41	*trnV-GAC*	GAU	D	753	1.6	*trnD-GUC*
GUC	V	163	0.51		GAC	D	187	0.4	
GUA	V	479	1.49		GAA	E	933	1.5	*trnE-UUC*
GUG	V	192	0.6	*trnV-UAC*	GAG	E	314	0.5	
UCU	S	499	1.63	*trnS-GGA*	UGU	C	198	1.49	*trnC-GCA*
UCC	S	302	0.99		UGC	C	67	0.51	
UCA	S	326	1.07		UGA	*	16	0.64	
UCG	S	214	0.7	*trnS-UGA*	UGG	W	400	1	*trnW-CCA*
CCU	P	334	1.37	*trnP-UGG*	CGU	R	312	1.25	*trnR-ACG*
CCC	P	205	0.84		CGC	R	119	0.48	*trnR-UCU*
CCA	P	276	1.13		CGA	R	327	1.31	
CCG	P	160	0.66		CGG	R	130	0.52	
ACU	T	507	1.63		AGA	R	450	1.8	
ACC	T	223	0.72		AGG	R	163	0.65	
ACA	T	365	1.17	*trnT-UGU*	AGU	S	385	1.26	*trnS-GCU*
ACG	T	149	0.48	*trnT-GGU*	AGC	S	106	0.35	
GCU	A	533	1.72	*trnA-UGC*	GGU	G	503	1.27	*trnG-GCC*
GCC	A	200	0.64		GGC	G	165	0.42	
GCA	A	363	1.17		GGG	G	339	0.85	
GCG	A	147	0.47		GGA	G	582	1.47	*trnG-UCC*

*indicates the stop codon.

### Repeat Analysis

**FIGURE 2:**
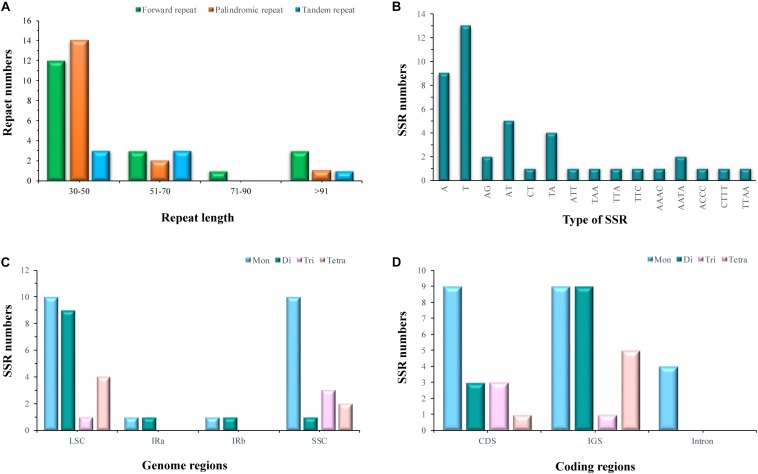
Statistics of repeat elements in the chloroplast genome of *Euphrasia regeli*. **(A)** Number of palindromic repeats, forward repeats and tandem repeats. **(B)** Frequency of identified SSR motifs. **(C)** Frequency of SSRs in the LSC, SSC, and IR. **(D)** Total numbers of identified SSRs in coding regions.

Of the *E. regelii* cp genome, 19 forward repeats, 17 palindromic repeats, and 7 tandem repeats were detected (Figure 2). More than half of the repeats (58.3%) were found in intergenic regions and introns, and 74.4% of these repeats have a repeat length between 30 and 50 bp (Figure 2). Within the CDS region, only two genes (*ycf1* and *ycf2*) contained six forward repeats, six palindromic repeats and two tandem repeats, respectively (Supplementary Tables S5, S6). A total of 44 SSRs were detected in the *E. regelii* cp genome, the majority of which were mononucleotide repeats (22), followed by dinucleotide (12), tetranucleotide (6), and trinucleotide (4) repeats. Most SSRs (29) were distributed in non-coding regions with the remaining 15 SSRs located in genic regions including *rpoC2*, *psbC*, *atpB*, *rpoA*, *ycf1*, *ccsA* (Figure 2). Just over half (54.5%) of the SSRs were located in the LSC region, whereas 36.4 and 9.1% were found in the SSC and the IR regions (Figure 2).

### Genome Comparison and Selective Pressure Analyses

**FIGURE 3:**
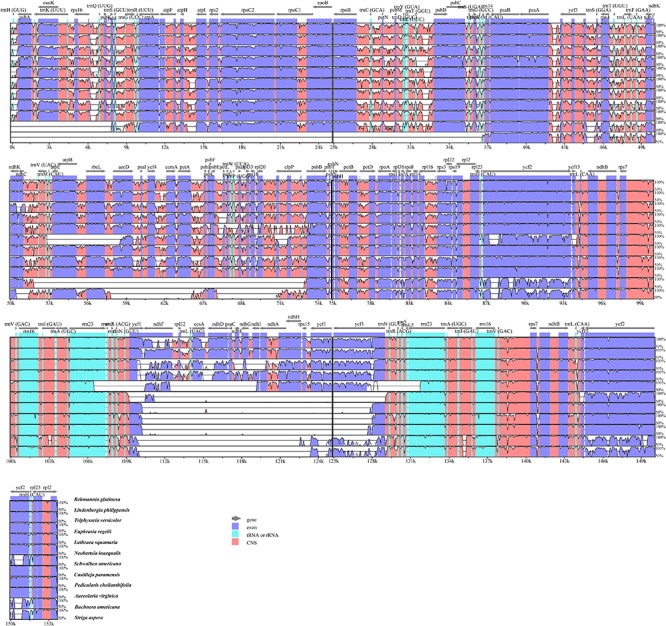
Percentages of identity comparing 12 chloroplast genomes from Orobanchaceae to the reference *Rehmannia glutinosa* (mVISTA). The y-axis represents the percent identity within 50–100%. Genome regions are color-coded as protein coding (purple), rRNA or tRNA coding genes (blue), and non-coding sequences (pink).

**FIGURE 4:**
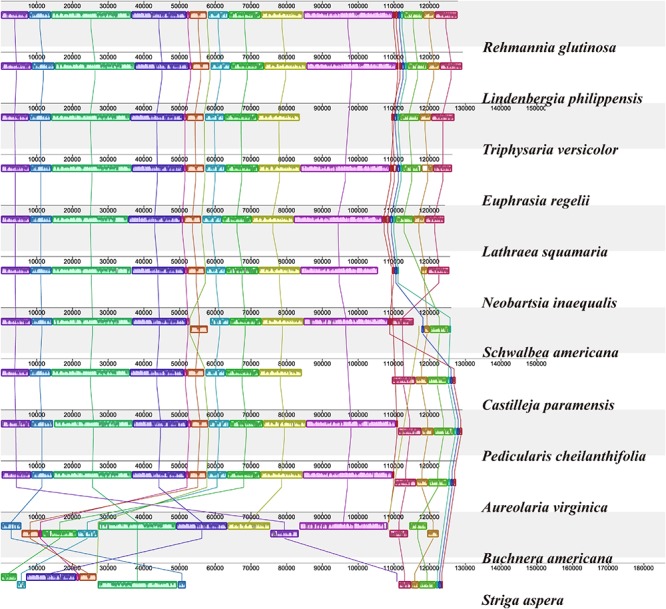
Plastome alignment of 12 species in the Orobanchaceae family. Alignment was carried out with only one copy of the IR taken out in Mauve v1.1.1 using *R. glutinosa* as a reference. Blocks on the top row are in the same orientation, while blocks on the bottom row are in inverse orientation.

**FIGURE 5:**
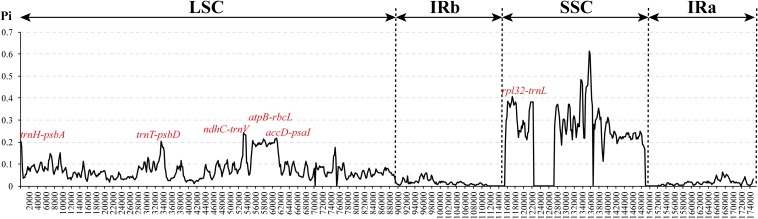
Nucleotide diversity (Pi) in the complete cp genomes of four Orobanchaceae species (*E. regelii*, *Lathraea squamaria, Neobartsia inaequalis*, and *Schwalbea americana*). Sliding window analysis with a window length of 600 bp and a step size of 200 bp.

To investigate cp genome divergence between *E. regelii* and other Orobanchaceae species, sequence alignment of 12 cp genomes were conducted using the annotated cp genome of *R. glutinosa* as a reference. The results indicated that the IR regions are more conserved than the SC regions and that the divergence in intergenic regions is higher than in genic regions (Figure 3). Many differences were found in the SSC regions of these plastomes, and the LSC regions of *B. americana* and *S. aspera* differed markedly from other autotrophic and parasitic species (Figure 4). The cp genome of *E. regelii* is very similar to the plastomes of *R. glutinosa*, *L. philippensis*, *T. versicolor*, *L. squamaria*, and *N. inaequalis*. All other plastomes contained multiple rearrangements, especially in *B. americana* and *S. aspera*. No rearrangements were detected in the three included Rhinantheae species (*E. regelii*, *L. squamaria, N. inaequalis*) except that some genes within the SSC region of *N. inaequalis* were lost. However, the orientation of the SSC region of *S. americana* was inverted and showed a reverse gene order compared to the other three Rhinantheae species. A sliding window analysis indicated that most of the variation in the cp genomes of the three Rhinantheae species occurred in the LSC and SSC regions (Figure 5). The most divergent non-coding regions among the four Rhinantheae cp genomes were *trnH* (*GUG*) *– psbA*, *rps16 – trnQ* (*UUG*), *trnS* (*GCU*) *– trnG* (*UCC*), *atpH-atpI*, *petN – psbM*, *trnT* (*GGU*) *– psbD*, *ndhC – trnV* (*UAC*), *rbcL – accD*, *petA – psbJ*, *clpP – psbB*, *ndhF – rpl32*, *rpl32 – trnL* (*UAG*). Although coding regions were conserved in these cp genomes, minor sequence variation was observed among the four cp genomes in the *rpoC2*, *rpoC1*, *ndhF*, *ycf1*, and *ycf2* gene.

**FIGURE 6:**
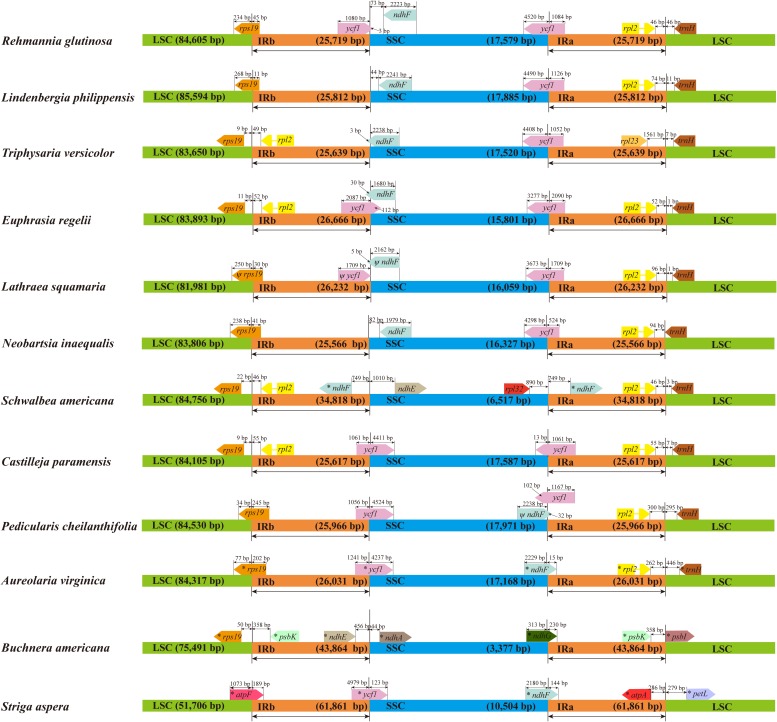
Chloroplast genome borders in 12 Orobanchaceae species. LSC (large single copy region), SSC (small single copy region), and IR (inverted repeat region). Ψ indicates a pseudogene. * indicates a region similar to a plastid gene.

Genomic structure and size varied in the 12 Orobanchaceae cp genomes and the IR/SC border regions of these species were also different (Figure 6). Fifteen genes including *rps19*, *ycf1*, *rpl2*, *ndhF*, *ndhE*, *rpl23*, *rpl32*, *psbK*, *ndhA*, *ndhG*, *atpF*, *atpA*, *psbI*, *petL* and *trnH*, were found in the LSC/IR and SSC/IR borders of the 12 plastomes. Of these, *S. aspera*, *B. americana*, and *S. americana* all exhibited larger plastome sizes due to the increased IR length, and the corresponding genes distributed in the SSC/IR border were quite different from other plastomes. Apart from the above three cp genomes, the IRs of *E. regelii* were much longer than in other cp genomes, especially in the area of the LSC/IRb and the IRb/SSC regions (Figure 6). The *ndhF* 3′-end sequence in the cp genomes of *E. regelii* and *L. squamaria* shared the region in the IRb with the rest of the *ycf1* 3′-end sequence, while the IRb-SSC border of *L. philippensis*, *R. glutinosa*, and *N. inaequalis*

were separated from the stop codon of *ndhF* by 32, 73, and 82 bp, respectively. Notably, genes located at the IR/SSC border of *Castilleja paramensis*, *P. cheilanthifolia*, and *A. virginica* showed a reverse gene order compared to *E. regelii.*

In order to detect whether the protein-coding genes of four Rhinantheae cp genomes (*E. regelii*, *L. squamaria, N. inaequalis*, and *S. americana*) were under selective pressure, rates of synonymous (dS) and non-synonymous (dN) substitutions, and the dN/dS ratios were calculated. As many pseudogenes were found in the cp genomes of *E. regelii* and *L. squamaria*, only 64 cp genes could be used for this analysis. The average dS values between paired Rhinantheae species (*E. regelii*-*S. americana*/*E. regelii*-*L. squamaria*/*S. americana*-*L. squamaria/E. regelii-N. inaequalis/S. americana-N. inaequalis/L. squamaria-N. inaequalis*) were 0.2175/0.1006/0.2131/0.0781/0.1861/0.0711 and the dN values ranged from 0 to 1.1435, with an average of 0.0718/0.0224/0.0857/0.0113/0.0639/0.0146, respectively (Supplementary Table S7). 305 paired dN/dS values were obtained most of which were less than 1, indicating that cp genes were under purifying selection. Only three genes (*clpP, ycf2, rps14*) had dN/dS values > 1, indicating that these genes had undergone positive selection.

### Phylogenetic Analyses Based on Chloroplast Genome Sequence

**FIGURE 7:**
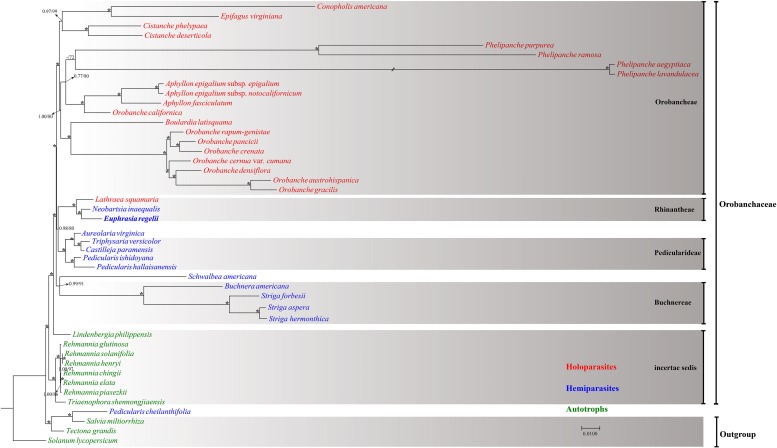
Maximum Likelihood tree based on the complete chloroplast genome. Numbers or asterisks above branches are statistical support values for maximum likelihood/Bayesian inference analyses, respectively, with * denoting maximum support in both analyses.

Forty-five complete chloroplast genomes were used to infer the phylogenetic position of *E. regelii* (Supplementary Table S1). Phylogenetic analyses were performed using Maximum likelihood (ML) and Bayesian inference (BI) with *Salvia miltiorrhiza*, *Tectona grandis*, and *Solanum lycopersicum* as outgroup. Two datasets were used to infer phylogenetic relationships, one dataset included the complete cp genome and the other dataset only TMCRs of the 45 cp genomes. Both datasets yielded a consistent phylogenetic signal (Figure 7 and Supplementary Figure S1) Except for *P. cheilanthifolia*, which clustered with the outgroup, all other species of the Orobanchaceae formed a monophyletic group with high bootstrap and BI support. Similarly, *E. regelii* and two other Rhinantheae species (*L. squamaria* and *N. inaequalis*) formed a highly supported clade, with *E. regelii* being sister to *N. inaequalis*. Unexpectedly, *S. americana*, another species in the Rhinantheae tribe, clustered with *Buchnera* and *Striga*. Apart from *L. squamaria*, all holoparasitic species clustered in the same clade which also was the most derived in Orobanchaceae. Autotrophic genera including *Lindenbergia* and *Rehmannia* belonged to the earliest diverging groups, suggesting that autotrophic lineages may be the ancestors of parasitic lineages in Orobanchaceae.

The phylogenetic relationship of 39 *Euphrasia* species was infered based on three cpDNA makers. All *Euphrasia* species formed a highly supported clade, however, species relationships remained unresolved (Supplementary Figure S2).

## Discussion

Here we present the complete chloroplast genome of *E. regelii* which is the first complete plastome for this hemiparasitic genus. The chloroplast genome of *E. regelii* displays the typical quadripartite structure with a LSC and a SSC region which are separated by two inverted repeat regions. The structure is comparable to the one of other hemiparasitic species in Orobanchaceae (Wicke et al., 2013, 2016; Cho et al., 2018). In the plastome of *E. regelii*, only 71 protein-coding genes are retained due to pseudogenization of some plastid genes, especially *ndhA – E, ndhG – K*, and *ycf*15. Previous studies indicated that relaxed selective constraints in relation to photosynthesis resulted in extensive pseudogenization of *ndh* genes in some parasitic genera such as *Lathraea*, *Pedicularis*, and *Schwalbea* (Barrett et al., 2013; Wicke et al., 2013; Cho et al., 2018). In contrast, the *ycf*15 gene is usually truncated as a pseudogene in many angiosperm chloroplast genomes (Dong et al., 2013; Fajardo et al., 2013; Hu et al., 2016; Lu et al., 2016; Ge et al., 2018). Gene loss or plastome reduction is a common phenomenon in most parasitic plant species (Wicke et al., 2013; Samigullin et al., 2016), however, this was not observed in the cp genome of *E. regelii* as *ndh* genes were only pseudogenized but not lost. It has been shown that *ndh* genes were pseudogenized or lost entirely several times during land plant evolution, which is largely related to a heterotrophic lifestyle (Wicke et al., 2011; Barrett and Davis, 2012; Barrett et al., 2014; Graham et al., 2017; Wicke and Naumann, 2018). *Euphrasia* species are facultative hemiparasites which can complete their lifecycle without a host, however, they grow much better attached to a suitable host (Twyford et al., 2019). This facultative lifestyle probably accounts for the retention and subsequent pseudogenization of *ndh* genes. Similar to most angiosperm cp genomes, the overall GC content and the codon usage of *E. regelii* cp genome is heavily biased.

Repeat elements in plastomes were shown to play an important role in genomic rearrangements and recombination (Asano et al., 2004; Weng et al., 2013). Low number of repeat elements were found in the cp genome of *E. regelii* compared to the previously published *Rehmannia* plastome (Zeng et al., 2017). Most repeats were located in intergenic regions or *ycf* genes (*ycf1* and *ycf2*) which is similar to the situation in other angiosperm lineages (Curci et al., 2015; Yang et al., 2016; Zhou et al., 2016). Chloroplast simple sequence repeats (cpSSRs) have been proven to be an important molecular marker for distinguishing species at lower taxonomic levels, and are therefore potentially useful marker for population genetics (Provan et al., 2001; Yang et al., 2011; Xue et al., 2012; Hu et al., 2016; Ruhsam et al., 2016). In the present study, 44 SSRs were detected in the *E. regelii* cp genome with mononucleotide repeats (A/T) being the most abundant type. Poly (A)/(T) SSRs are usually more common than other SSR repeat types in many plant cp gnomes (Yang et al., 2016; Asaf et al., 2017b; Dong et al., 2017; Li et al., 2018; Wang et al., 2018b; Ye et al., 2018; Zhou et al., 2018). Likewise, most cpSSRs were observed in non-coding regions, and only a small proportion was found in coding regions. CpSSRs located in non-coding regions are generally short mononucleotide tandem repeats and commonly show intraspecific variation in repeat numbers (Eguiluz et al., 2017). Therefore, cpSSR loci detected in this study will be useful tools for investigating levels of genetic diversity in *Euphrasia* and might even be able to discriminate between species.

Morphological similarity renders the reliable identification of many *Euphrasia* species challenging. In addition, standard DNA plant barcodes (Techen et al., 2014; Li et al., 2015) have failed to discriminate between *Euphrasia* species (Wang et al., 2018a). Therefore, it is necessary to develop *Euphrasia* specific DNA barcodes. Here, several highly variable cpDNA markers were obtained based on the comparative chloroplast genome analyses of Orobanchaceae species which could be tested as *Euphrasia* specific DNA barcodes. These regions might also provide sufficient genetic variation for resolving the phylogenetic relationships between *Euphrasia* species. Compared to two photoautotrophic species our results indicated that there are no structural rearrangements in the cp genome of *E. regelii* which is probably related to the facultative hemiparasitic life form of this species (Frailey et al., 2018). No major gene rearrangements were detected among four Rhinantheae species, except for *B. americana* which had a reversed SSC region. The SSC region is usually flipped in plastomes and the reversed SSC often show in a 50:50 ratio in plant cells (Palmer, 1985; Frailey et al., 2018). A similar situation was also detected in *A. virginica* and two other Pedicularideae species.

Size variability in cp genomes is usually due to the contraction and expansion of the IRs (He et al., 2017). This was apparent in the plastomes of *S. aspera*, *B. americana*, and *S. americana* where the IRs were much longer. Interestingly, the cp IR borders of *B. americana* are quite different from other Rhinantheae species as most of the repeat region extended into the SSC region. A previous study showed that *B. americana* belongs to an early diverging lineage in the Rhinantheae clade (McNeal et al., 2013), which suggests that the repeat expansion occurred independently in the *B. americana* lineage. The IR length of *E. regelii* was the longest out of the other three above cp genomes sequenced and was expanded much more than cp genomes of *L. squamaria* and *N. inaequalis*. Generally, *ycf1* in the IRb is often pseudogenized in several angiosperm cp genomes (Daniell et al., 2006; Yao et al., 2016). However, no internal stop codons were detected in the coding sequence of *ycf1* in *E. regelii*, thus the additional length of *ycf1* affected the IR length and the gene distribution at the SC/IR borders. We hypothesize that the expansion of the IR caused a duplication of *ycf1*, like it has been reported for *Eucommia ulmoides* and *Fagopyrum dibotrys* (Wang et al., 2016, 2018b).

The results from the sequence divergence analysis of protein coding genes in four Rhinantheae plastomes indicated low sequence divergence and purifying selection (dN/dS < 1) for most genes which is consistent with the results from other studies (Rousseau-Gueutin et al., 2015; Xu et al., 2015; Zhou et al., 2016; Yin et al., 2018). Only three protein-coding genes (*clpP, ycf2, rps14*) were under positive selection. *ClpP*, which encodes a proteolytic subunit of the ATP-dependent protease, is very important for chloroplast biogenesis (Shikanai et al., 2001). *Clp* proteases are highly conserved in many organisms (Schirmer et al., 1996; Shikanai et al., 2001) but previous studies indicated that *clpP* genes showed significantly accelerated substitution rates and were under positive selection in *Pelargonium* plastid genomes (Weng et al., 2017). It is likely that *clpP* may have higher substitution rates in parasitic plant species. *Ycf2* is one of the largest genes encoding for a putative membrane protein (Drescher et al., 2000; Kikuchi et al., 2013) and has rapidly evolved in several species of *Fagopyrum*, *Ipomoea*, *Ophrys*, and Mimosoideae (Cho et al., 2015; Mensous et al., 2017; Park et al., 2018; Roma et al., 2018). Likewise, *ycf2* may have evolved at a faster rate in the Rhinantheae plastomes.

Chloroplast genomes which contain sufficient informative sites have been proven to be effective in resolving phylogenetic relationships among angiosperms even at lower taxonomic levels (Ma et al., 2014; Carbonell-Caballero et al., 2015; Yang et al., 2016; Dong et al., 2017; Zhang et al., 2017; Zhao et al., 2018). We retrieved the available cp genomes of non-parasitic (autotrophic) and parasitic species in the Orobanchaceae and inferred the phylogeny of Orobanchaceae based on ML and Bayesian methods. Our results were consistent with the results of previous studies based on nuclear and plastid markers (McNeal et al., 2013) as well as 17 cp genomes (Samigullin et al., 2016). Except for the placement of *P. cheilanthifolia*, all the parasitic species formed a highly supported clade. Unexpectedly, the overall genomic structure of *P. cheilanthifolia* is more similar to the cp genome of autotrophic species than to that of the closely related *Pedicularis* species. Thus, high sequence divergence of the *P. cheilanthifolia* plastome resulted in a discordant phylogenetic position. Previous phylogenetic analyses based on a few cpDNA markers did not support the monophyly of Rhinantheae (Olmstead et al., 2001) which is consistent with our results of four Rhinantheae species where *S. americana* was not included in Rhinantheae but was sister to Buchnereae. Also, *Euphrasia* was more closely related to *Neobartsia* than to *Lathraea* which is consistent with previous phylogenetic studies in the Rhinantheae tribe (McNeal et al., 2013; Pinto-Carrasco et al., 2017). Our results suggested that all non-parasitic species belonged to the earliest diverging lineages in Orobanchaceae indicating that autotrophy was the ancestral state in this mainly parasitic family. This has also been highlighted by previous studies (Bennett and Mathews, 2006; McNeal et al., 2013). However, to obtain a reliable inference of ancestral states a comprehensive sampling of all taxa in Orobanchaceae is necessary as limited taxon sampling can result in different tree topologies (Leebens-Mack et al., 2005; Eguiluz et al., 2017).

Due to the recent divergence of many *Euphrasia* species (Gussarova et al., 2008), the commonly used standard DNA barcodes are not variable enough to resolve phylogenetic relationships in *Euphrasia* which is obvious from our results based on three cpDNA fragments as well as previous phylogenetic studies (Gussarova et al., 2008; Wang et al., 2018a). However, even the complete chloroplast genome might not substantially raise species discriminatory power in evolutionarily young lineages, and very large numbers of characters from the nuclear genome are likely to be required for this task (Ruhsam et al., 2015).

## Conclusion

The complete chloroplast genome of *E. regelii*, which is the first published cp genome in *Euphrasia*, provides a valuable genomic resource for this important medicinal plant and other *Euphrasia* species. The structure and gene content of the cp genome are comparable to other hemiparasitic and two photoautotrophic species in Orobanchaceae. No structural rearrangements were detected, however, 10 genes encoding the NAD(P)H dehydrogenase complex were widely pseudogenized but not lost. Coding gene sequence divergence analyses indicated that only three plastid genes were under positive selection. We also identified cpSSRs that could be used for population genetic studies in *Euphrasia* and whole cp genome comparison of *E. regelii* with other Orobanchaceae species indicated several variable hotspots, which could be used to develop DNA markers suitable for the discrimination between *Euphrasia* species, and for the inference of phylogenetic relationships.

## Author Contributions

XW and TZ conceived and designed the experiments. JW, WL, YX, HZ, and FX performed the experiments and analyzed the data. TZ, XZ, XW, and MR wrote the manuscript. All authors read and approved the final manuscript.

## Conflict of Interest Statement

The authors declare that the research was conducted in the absence of any commercial or financial relationships that could be construed as a potential conflict of interest.

## Footnotes

1 https://treebase.org

## Supplementary Material

FIGURE S1 Phylogenetic relationship inferred from Maximum Likelihood/Bayesian Inference analysis based on the most conserved regions (TMCRs) of the chloroplast genome. The numbers associated with each node are bootstrap support and posterior probability values, respectively. Asterisks indicate support values of 100/1.0.

FIGURE S2 Phylogenetic relationships of *Euphrasia* using cpDNA *trn*L intron, *trn*L-*trn*F, and *atp*B-*rbc*L. **(A)** Phylogenetic tree inferred from ML analysis. **(B)** Phylogenetic tree inferred from BI analysis. The numbers associated with each node are bootstrap support and posterior probability values.

TABLE S1 List of plastome sequences included in the phylogenetic analyses.

TABLE S2 Primers used for Sanger re-sequencing.

TABLE S3 Models in ML and BI analysis based on different datasets.

TABLE S4 Genes encoded in the *Euphrasia regelii* chloroplast genome.

TABLE S5 Forward and Palindromic repeats in the *Euphrasia regelii* chloroplast genome.

TABLE S6 Tandem repeats in the *Euphrasia regelii* chloroplast genome.

TABLE S7 dN/dS ratio between pairwise of four Rhinantheae species protein coding sequences.
