# Supplementary material for: The Complete Chloroplast Genome of Euphrasia regelii, Pseudogenization of ndh Genes and the Phylogenetic Relationships Within Orobanchaceae
Source: Front Genet. 2019 May 14;10:444. doi: 10.3389/fgene.2019.00444 (PMC6528182; doi:10.3389/fgene.2019.00444)
Supplement: FIGURE S1 — Phylogenetic relationship inferred from Maximum Likelihood/Bayesian Inference analysis based on the most conserved regions (TMCRs) of the chloroplast genome. The numbers associated with each node are bootstrap support and posterior probability values, respectively. Asterisks indicate support values of 100/1.0. [file Data_Sheet_1.ZIP › Supplementary Materials/Table S1.docx]

**Table S1** List of plastome sequences included in the phylogenetic analyses

| No. | Family | Taxon | Accesion number |
| --- | --- | --- | --- |
| 1 | Orobanchaceae | *Boulardia latisquama* | NC_025641 |
| 2 | Orobanchaceae | *Castilleja paramensis* | NC_031805 |
| 3 | Orobanchaceae | *Cistanche deserticola* | NC_021111 |
| 4 | Orobanchaceae | *Cistanche phelypaea* | NC_025642 |
| 5 | Orobanchaceae | *Conopholis americana* | NC_023131 |
| 6 | Orobanchaceae | *Epifagus virginiana* | NC_001568 |
| 7 | Orobanchaceae | *Euphrasia regelii* | MK070895 |
| 8 | Orobanchaceae | *Lathraea squamaria* | NC_027838 |
| 9 | Orobanchaceae | *Lindenbergia philippensis* | NC_022859 |
| 10 | Orobanchaceae | *Neobartsia inaequalis* | KF922718 |
| 11 | Orobanchaceae | *Orobanche austrohispanica* | NC_031441 |
| 12 | Orobanchaceae | *Orobanche californica* | NC_025651 |
| 13 | Orobanchaceae | *Orobanche cernua* var*. cumana* | KT387722 |
| 14 | Orobanchaceae | *Orobanche crenata* | NC_024845 |
| 15 | Orobanchaceae | *Orobanche densiflora* | NC_031442 |
| 16 | Orobanchaceae | *Orobanche gracilis* | NC_023464 |
| 17 | Orobanchaceae | *Orobanche pancicii* | NC_031443 |
| 18 | Orobanchaceae | *Orobanche rapum-genistae* | NC_031444 |
| 19 | Orobanchaceae | *Pedicularis cheilanthifolia* | NC_036010 |
| 20 | Orobanchaceae | *Pedicularis hallaisanensis* | NC_037433 |
| 21 | Orobanchaceae | *Pedicularis ishidoyana* | NC_029700 |
| 22 | Orobanchaceae | *Phelipanche purpurea* | NC_023132 |
| 23 | Orobanchaceae | *Phelipanche ramosa* | NC_023465 |
| 24 | Orobanchaceae | *Rehmannia chingii* | NC_033534 |
| 25 | Orobanchaceae | *Rehmannia elata* | NC_034312 |
| 26 | Orobanchaceae | *Rehmannia glutinosa* | NC_034308 |
| 27 | Orobanchaceae | *Rehmannia henryi* | NC_034309 |
| 28 | Orobanchaceae | *Rehmannia solanifolia* | NC_034310 |
| 29 | Orobanchaceae | *Rehmannia piasezkii* | NC_034311 |
| 30 | Orobanchaceae | *Schwalbea americana* | NC_023115 |
| 31 | Orobanchaceae | *Aureolaria virginica* | MF780870 |
| 32 | Orobanchaceae | *Buchnera americana* | MF780871 |
| 33 | Orobanchaceae | *Striga aspera* | MF780872 |
| 34 | Orobanchaceae | *Striga forbesii* | MF780873 |
| 35 | Orobanchaceae | *Striga hermonthica* | MF780874 |
| 36 | Orobanchaceae | *Phelipanche aegyptiaca* | KU212370 |
| 37 | Orobanchaceae | *Phelipanche lavandulacea* | KU212371 |
| 38 | Orobanchaceae | *Triphysaria versicolor* | KU212369 |
| 39 | Orobanchaceae | *Triaenophora shennongjiaensis* | NC_039781 |
| 40 | Orobanchaceae | *Aphyllon fasciculatum* | NC_039679 |
| 41 | Orobanchaceae | *Aphyllon epigalium* subsp*. epigalium* | MH050785 |
| 42 | Orobanchaceae | *Aphyllon epigalium* subsp*. notocalifornicum* | MH050786 |
| 43 | Lamiaceae | *Salvia miltiorrhiza* | NC_020431 |
| 44 | Lamiaceae | *Tectona grandis* | NC_020098 |
| 45 | Solanaceae | *Solanum lycopersicum* | NC_007898 |
