# Supplementary material for: The Complete Chloroplast Genome of Euphrasia regelii, Pseudogenization of ndh Genes and the Phylogenetic Relationships Within Orobanchaceae
Source: Front Genet. 2019 May 14;10:444. doi: 10.3389/fgene.2019.00444 (PMC6528182; doi:10.3389/fgene.2019.00444)
Supplement: FIGURE S1 — Phylogenetic relationship inferred from Maximum Likelihood/Bayesian Inference analysis based on the most conserved regions (TMCRs) of the chloroplast genome. The numbers associated with each node are bootstrap support and posterior probability values, respectively. Asterisks indicate support values of 100/1.0. [file Data_Sheet_1.ZIP › Supplementary Materials/Table S4.docx]

**Table S4** Genes encoded in the *Euphrasia regeli* chloroplast genome

| Gene categories | Gene groups | Gene names |
| --- | --- | --- |
| Genes for photosynthesis | PhotosystemⅠ | *psaA, psaB, psaC, psaI, psaJ* |
|  | Photosystem II | *psbA, psbB, psbC, psbD, psbE, psbF, psbH, psbI, psbJ, psbK, psbL, psbM, psbN, psbT, psbZ* |
|  | Subunits of cytochrome | *petA, petB^a^, petD^a^, petG, petL, petN* |
|  | Subunits of ATP synthase | *atpA, atpB, atpE, atpF^a^, atpH, atpI* |
|  | Subunits of NADH dehydrogenase | *ndhA^Ψ^, ndhB^Ψ^*^,c^*, ndhC^Ψ^, ndhD^Ψ^, ndhE^Ψ^, ndhF, ndhG^Ψ^, ndhH^Ψ^, ndhI^Ψ^, ndhJ^Ψ^, ndhK^Ψ^* |
|  | Large subunit of Rubisco | *rbcL* |
| Self-replication | Ribosomal protein (small subunit) | *rps2, rps3, rps4, rps7^c^, rps8, rps11, rps12^a,b^, rps14, rps15, rps16^a,^, rps18, rps19* |
|  | Ribosomal protein (large subunit) | *rpl2^a,c^, rpl14, rpl16^a,c^, rpl20, rpl22, rpl23^c^, rpl32^c^, rpl33, rpl36* |
|  | DNA-dependent RNA polymerase | *rpoA, rpoB, rpoC1^a^, rpoC2* |
|  | Transfer RNAs | *trnA-UGC^a,c^, trnC-GCA, trnD-GUC, trnE-UUC, trnfM-CAU, trnF-GAA, trnG-GCC, trnG-UCC^a^, trnH-CAU^c^ trnH-GUG, trnI-GAU^a,c^, trnK-UUU^a^, trnL-CAA^c^, trnL-UAG, trnL-UAA^a^, trnM-CAU, trnN-GUU^c^, trnP-UGG, trnQ-UUG, trnR-ACG^c^, trnR-UCU, trnS-GGA, trnS-GCU, trnS-UGA, trnT-GGU, trnT-UGU, trnV-GAC^c^, trnV-UAC^a^, trnW-CCA, trnY-GUA* |
|  | Ribosomal RNAs | *rrn4.5^c^, rrn5^c^, rrn16^c^, rrn23^c^* |
| Other genes | Translation-related gene | *infA* |
|  | membrane protein | *cemA* |
|  | Acetyl-CoA carboxylase gene | *accD* |
|  | ATP-dependent protease subunit | *clpP^b^* |
|  | Maturase | *matK* |
|  | c-type Cytochrome biogenesis | *ccsA* |
|  | Conserved hypothetical chloroplast reading frames | *ycf1^b^, ycf2^b^, ycf3^a^, ycf4, ycf15^b,Ψ^* |

^a^ containing one intron; ^b^ containing two introns; ^c^ Genes located in the IR regions; *^Ψ^* pseudogenes
