# Supplementary figures and images for: The Complete Chloroplast Genome of Euphrasia regelii, Pseudogenization of ndh Genes and the Phylogenetic Relationships Within Orobanchaceae
Source: Front Genet. 2019 May 14;10:444. doi: 10.3389/fgene.2019.00444 (PMC6528182; doi:10.3389/fgene.2019.00444)

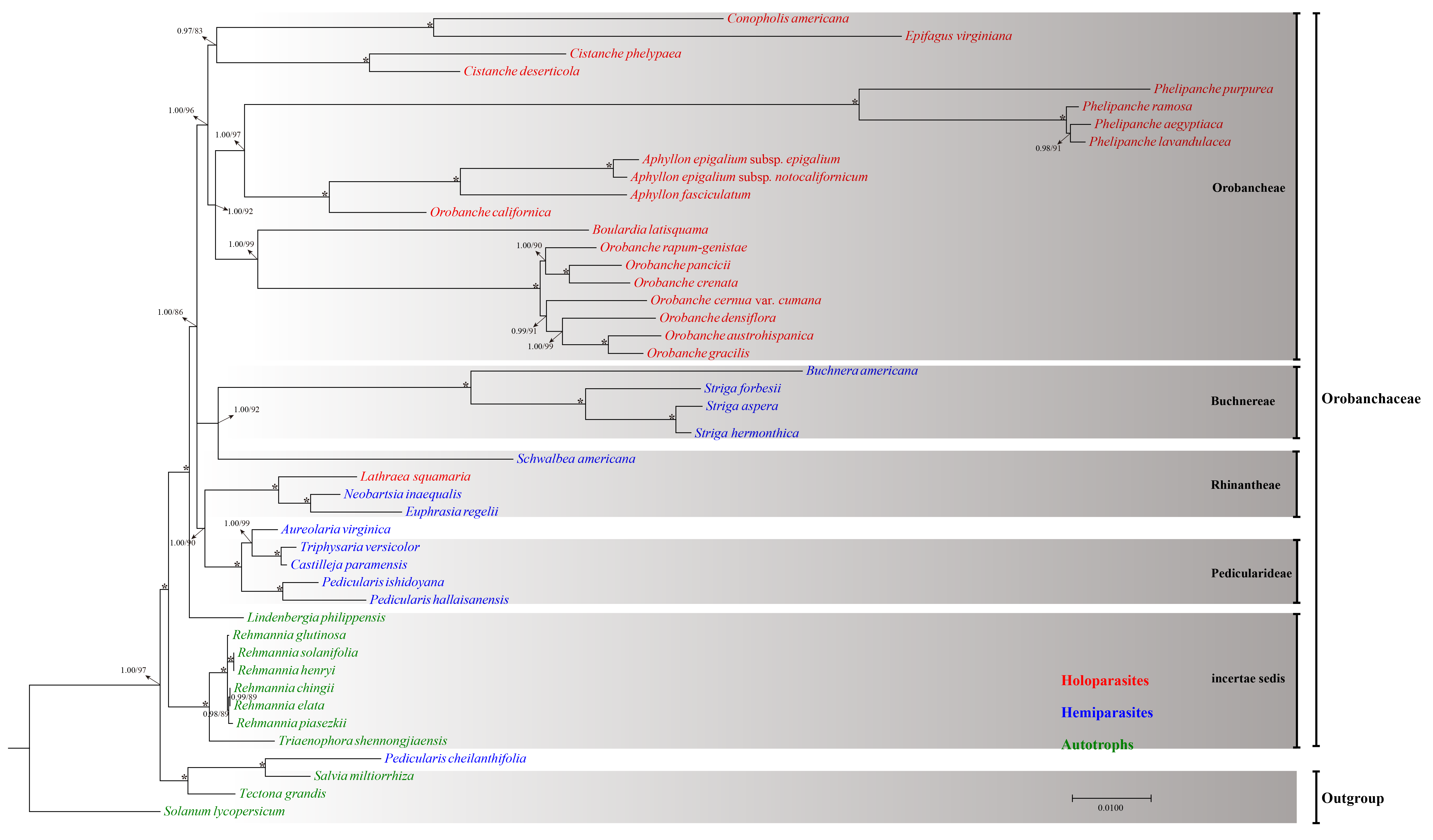

Supplement: FIGURE S1 — Phylogenetic relationship inferred from Maximum Likelihood/Bayesian Inference analysis based on the most conserved regions (TMCRs) of the chloroplast genome. The numbers associated with each node are bootstrap support and posterior probability values, respectively. Asterisks indicate support values of 100/1.0. [file Data_Sheet_1.ZIP › Supplementary Materials/Fig. S1.png]

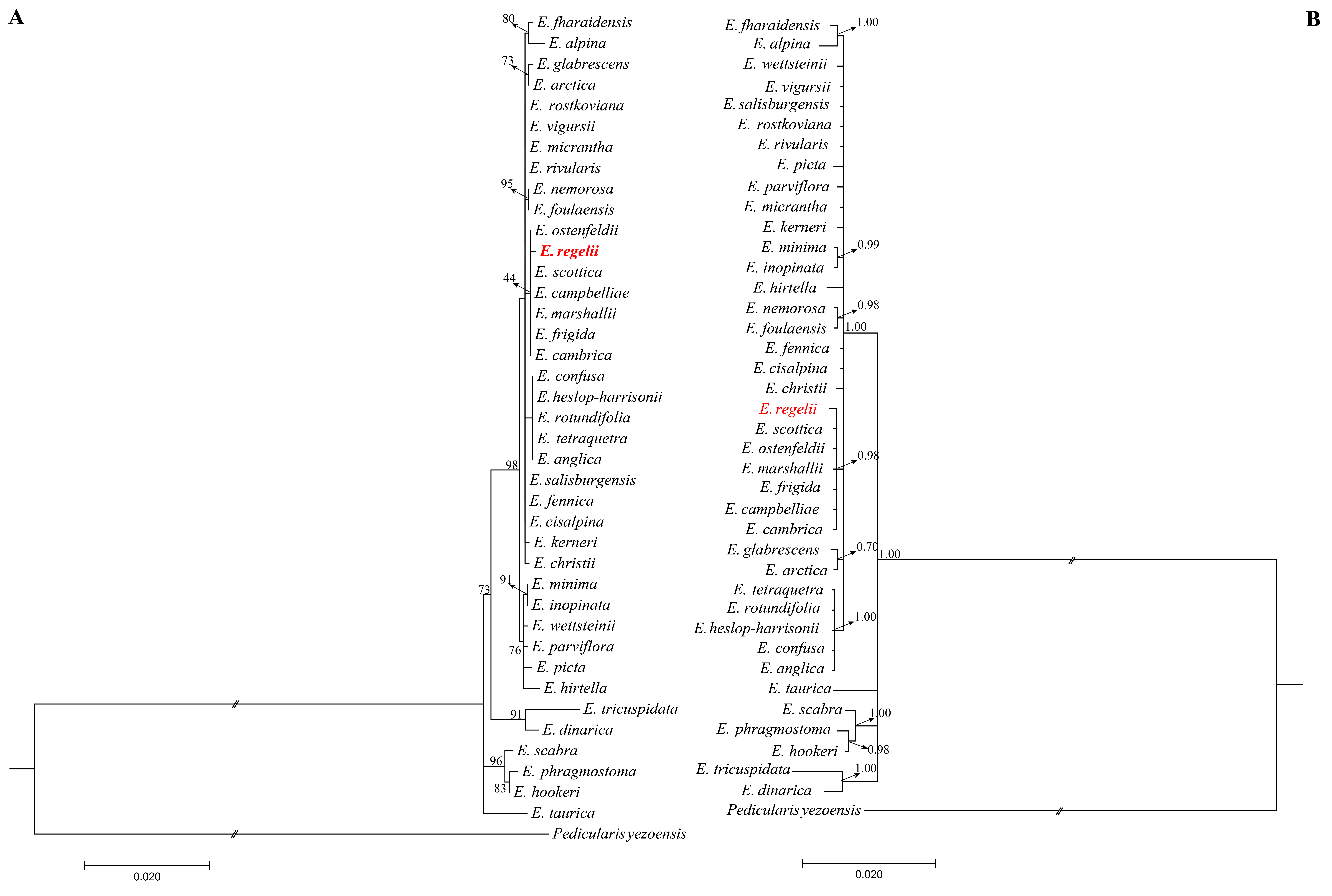

Supplement: FIGURE S1 — Phylogenetic relationship inferred from Maximum Likelihood/Bayesian Inference analysis based on the most conserved regions (TMCRs) of the chloroplast genome. The numbers associated with each node are bootstrap support and posterior probability values, respectively. Asterisks indicate support values of 100/1.0. [file Data_Sheet_1.ZIP › Supplementary Materials/Fig. S2.jpg]
